# Enhanced and Extended Anti-Hypertensive Effect of VP5 Nanoparticles

**DOI:** 10.3390/ijms17121977

**Published:** 2016-11-25

**Authors:** Ting Yu, Shengnan Zhao, Ziqiang Li, Yi Wang, Bei Xu, Dailong Fang, Fazhan Wang, Zhi Zhang, Lili He, Xiangrong Song, Jian Yang

**Affiliations:** 1 School of Applied Chemistry and Biological Technology, Shenzhen Polytechnic, Shenzhen 518055, China; tingyu419@126.com; 2 College of Pharmacy, Southwest University for Nationalities, Chengdu 610041, China; zhaoshn11@163.com (S.Z.); lilihes@163.com (L.H.); 3 State Key Laboratory of Biotherapy and Cancer Center, West China Hospital, and Collaborative Innovation Center for Biotherapy, Sichuan University, Chengdu 610041, China; ziqiangli21@gmail.com (Z.L.); xb1990625@126.com (B.X.); fangdailongtwozero@126.com (D.F.); FazhanWang_16@163.com (F.W.); zhangzhi02@gmail.com (Z.Z.); 4 Department of Medical Oncology, Dana-Farber Cancer Institute and Harvard Medical School, Boston, MA 02115, USA; Yi_Wang@dfci.harvard.edu

**Keywords:** PLGA nanoparticles, antihypertensive peptide, oral administration, sustained release, continuously antihypertensive effect

## Abstract

Hypertension has become a significant global public health concern and is also one of the most common risk factors of cardiovascular disease. Recent studies have shown the promising result of peptides inhibiting angiotensin converting enzyme (ACE) in lowering the blood pressure in both animal models and humans. However, the oral bioavailability and continuous antihypertensive effectiveness require further optimization. Novel nanoparticle-based drug delivery systems are helpful to overcome these barriers. Therefore, a poly-(lactic-*co*-glycolic) acid nanoparticle (PLGANPs) oral delivery system, of the antihypertensive small peptides Val-Leu-Pro-Val-Pro (VLPVP, VP5) model, was developed in this study and its antihypertensive effect was investigated in spontaneously hypertensive rats (SHRs) for the first time. The obtained VP5 nanoparticles (VP5-NPs) showed a small particle size of 223.7 ± 2.3 nm and high entrapment efficiency (EE%) of 87.37% ± 0.92%. Transmission electronic microscopy (TEM) analysis showed that the nanoparticles were spherical and homogeneous. The optimal preparation of VP5-NPs exhibited sustained release of VP5 in vitro and a 96 h long-term antihypertensive effect with enhanced efficacy in vivo. This study illustrated that PLGANPs might be an optimal formulation for oral delivery of antihypertensive small peptides and VP5-NPs might be worthy of further development and use as a potential therapeutic strategy for hypertension in the future.

## 1. Introduction

Hypertension (high blood pressure), as a global public health risk, is highly associated with heart disease, stroke, kidney failure, premature death and disability [1]. Currently, many anti-hypertensive therapeutic drugs have been developed and are now available for clinical treatment, including thiazide diuretics, beta-blockers, renin-angiotensin-aldosterone system (RAAS) inhibitors and calcium channel blockers. However, side effects have been reported with these treatments. Thiazide diuretics could potentially result in insulin resistance, dyslipidemia, and hyperuricemia, which would accelerate diabetes progression in patients with obesity and/or metabolic syndromes [2]. Traditional β-blockers appear to have a significantly higher risk of diabetes. RAAS inhibition, by an angiotensin converting enzyme (ACE) or an angiotensin receptor blocker (ARB) along with calcium channel blockers, appears to have improved safety properties, but some of them still have various side effects such as coughing, taste disturbances and skin rashes [3]. Clearly, future investigations for novel and safe antihypertensive drugs are still required.

Recently, a number of peptides inhibiting ACE have been demonstrated to reduce systolic (SBP) and diastolic blood pressure (DBP) in both animal models and humans [4]. Ile-Pro-Pro (IPP) and Val-Pro-Pro (VPP), the best characterized antihypertensive peptides, have been used as active ingredients in blood pressure control [5]. However, the effectiveness and the active duration of treatment are greatly compromised by their poor oral bioavailability. The major challenge is the peptide degradation in the gastrointestinal (GI) tract and poor permeation [6]. To overcome these barriers, peptide structural modifications, enzyme inhibitors, absorption enhancers, multifunctional polymers and different carrier systems have also been exploited to prevent proteolysis and to enhance systemic uptake. Among these different strategies, nanoparticles encapsulation is attractive due to its protective effect against degradation, enhanced uptake and sustained release [7].

Poly-(lactic-*co*-glycolic) acid (PLGA) is a well-known bio-degradable and biocompatible polymer approved by Food and Drug Administration (FDA) and European Medicines Agency (EMA) [8]. It has been used in a variety of biomedical devices and tissue engineering scaffolds, which are proven to be safe for clinical applications [9]. PLGA nanoparticles (PLGANPs) have gained great attention in the field of drug delivery [10,11]. Controllable release can be achieved by using PLGANPs to effectively protect encapsulated material from degradation in the GI tract [12,13,14]. However, due to the hydrophobic nature of the PLGA polymer it is challenging to achieve entrapment of hydrophilic peptides with high efficiency, especially with peptides of low molecular weight.

In this study, PLGANPs were investigated as a potential oral delivery system for the antihypertensive small peptides Val-Leu-Pro-Val-Pro (VLPVP, VP5) model. VP5 was previously prepared using deoxyribonucleic acid recombinant technology and its bioactivity had been proven by in vitro inhibitory activity of ACE (IC_50_ 1.8 µmol/L) and in vivo antihypertensive effect [15]. The formulation parameters of VP5 loaded PLGANPs (VP5-NPs) were systematically studied. The optimized VP5-NPs were then characterized in vitro and in vivo antihypertensive efficacy was analyzed with spontaneously hypertensive rats (SHRs).

## 2. Results

### 2.1. Optimization of Preparation Variables of VP5-NPs

**Figure 1:**
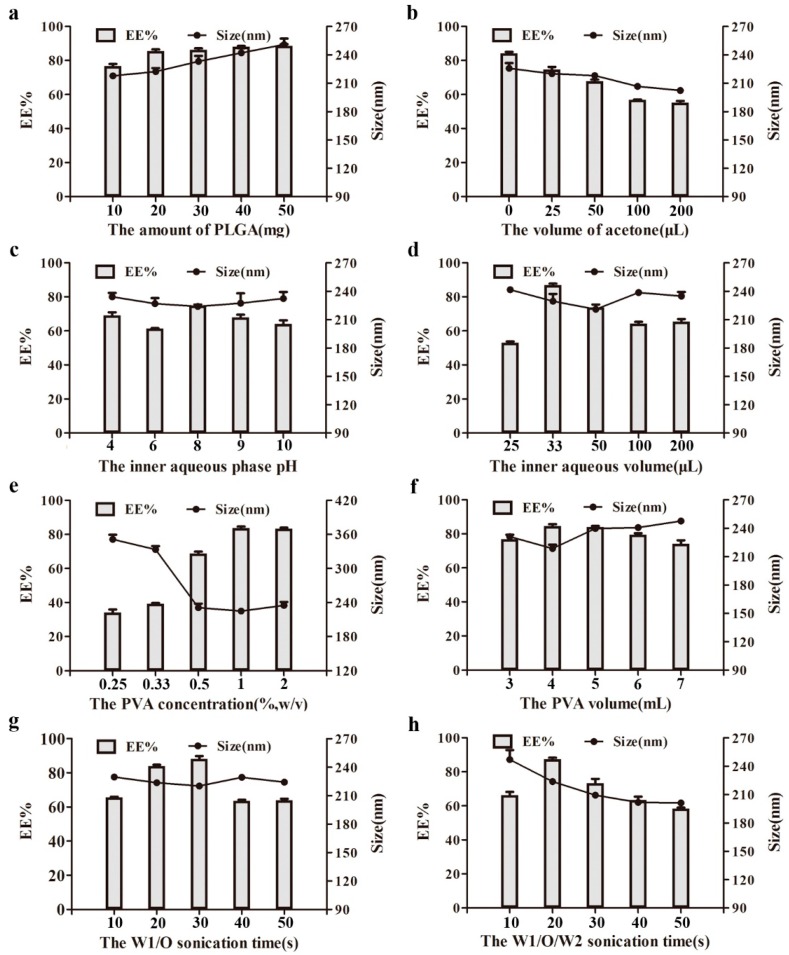
Effect of various processing parameters on the particle size (nm) and entrapment efficiencies (EE%) of nanoparticles, including the amount of poly-(lactic-*co*-glycolic) acid (PLGA) (**a**), the volume of acetone (**b**), the pH (**c**) and volume (**d**) of inner aqueous phase, the concentration (**e**) and volume (**f**) of poly (vinyl alcohol) (PVA), and the sonication time of W1/O (**g**) and W1/O/W2 (**h**). (*n* = 3).

VP5-NPs were successfully produced by systematically optimizing the factors that could affect the features of NPs, including the amount of PLGA, the volume of acetone, pH and the volume of inner aqueous phase, the concentration and volume of poly (vinyl alcohol) (PVA), and the sonication time. Firstly, the effects of the PLGA amount on entrapment efficiency (EE%), particle size, and particle distribution were investigated. As shown in Figure 1a, both the EE% and the particle size were positively correlated with the PLGA amount within the range of 10 to 50 mg. Figure 1b showed that the particle size decreased as the acetone increase. However, the EE% was significantly compromised by the presence of acetone. In Figure 1c, the particle size was not significantly affected by the pH of internal phase, and the EE% was observed to fluctuate as pH changed. As seen in Figure 1d, EE% of VP5 was significantly affected by the volume of inner aqueous phase, but negligibly by particle size.

The influence of the PVA concentration is illustrated in Figure 1e. As PVA concentration increased, the mean diameter first decreased dramatically and then stabilized over 0.5%. In contrast, the EE% of VP5-NPs exhibited an elevating trend with the increasing of PVA concentration. The effect of PVA volume on EE% and particle size were also studied and were similar to that of the PVA concentration. In Figure 1f, as the volume of PVA increased, the particle size was observed to decrease at low volume and then increase beyond 4 mL, while the EE% showed an opposite trend. In Figure 1g, as internal phase/organic phase (W1/O) sonication time was prolonged, the EE% increased rapidly first and then dropped, while particle size was relatively stable. Furthermore, the effect of the sonication time of internal phase / organic phase /external phase (W1/O/W2) on EE% and particle size was also explored. It was shown in Figure 1h that, the EE% was improved under short sonication, but was compromised once the sonication time was beyond 20 s, while the particle size decreased as the sonication time was prolonged.

Based on the above observations, the optimized preparation of VP5-NPs can be summarized as follows. A total of 20 mg of PLGA was dissolved in dichloromethane. The obtained organic phase was emulsified with 33 µL of VP5 solution by probe sonication at 80 W for 30 s in ice bath. The resulting primary emulsion was added to 4 mL of 1% (*w*/*v*) PVA solution dropwise. The mixture was sonicated for 20 s and then rotary evaporation under vacuum was used to remove dichloromethane at 37 °C to obt ain the nanoparticle suspension. Through ultracentrifugation at a speed of 50,000 rpm for 1 h to remove PVA and the unencapsulated VP5, the VP5-NPs were finally constructed.

### 2.2. Characterization of VP5-NPs

**Figure 2:**
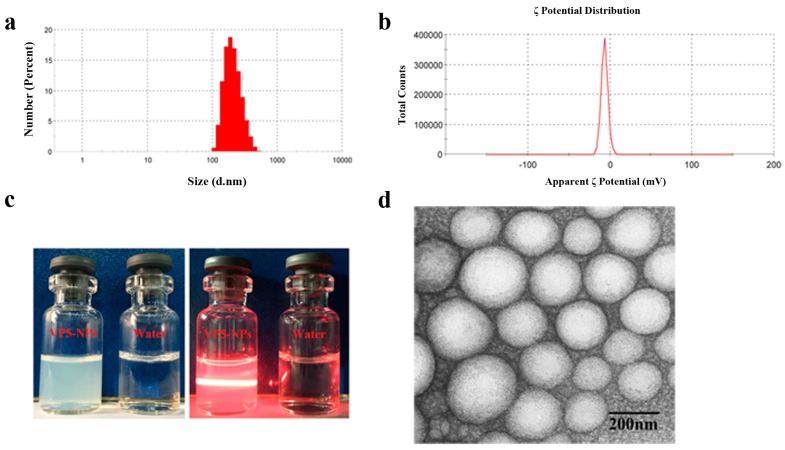
Pharmaceutical properties of VP5-NPs. (**a**) Size distribution; (**b**) ζ potential; (**c**) The appearance and Tyndall effect of VP5-NPs; (**d**) TEM image of VP5-NPs.

Overall, the optimal VP5-NPs were demonstrated to significantly improve the drug incorporation with an EE% of 87.37% ± 0.92% and drug loading capacity (DL%) of 2.10% ± 0.17%. The average particle size was 223.7 ± 2.3 nm (Figure 2a) with a narrow size distribution (PDI = 0.12 ± 0.01). The colloidal solution was observed as slightly blue opalescence with strong Tyndall effect (Figure 2c). As shown in the transmission electronic microscopy (TEM) image in Figure 2d, VP5-NPs were generally spherical and homogeneous, which was in good agreement with the narrow particle size distribution.

**Figure 3:**
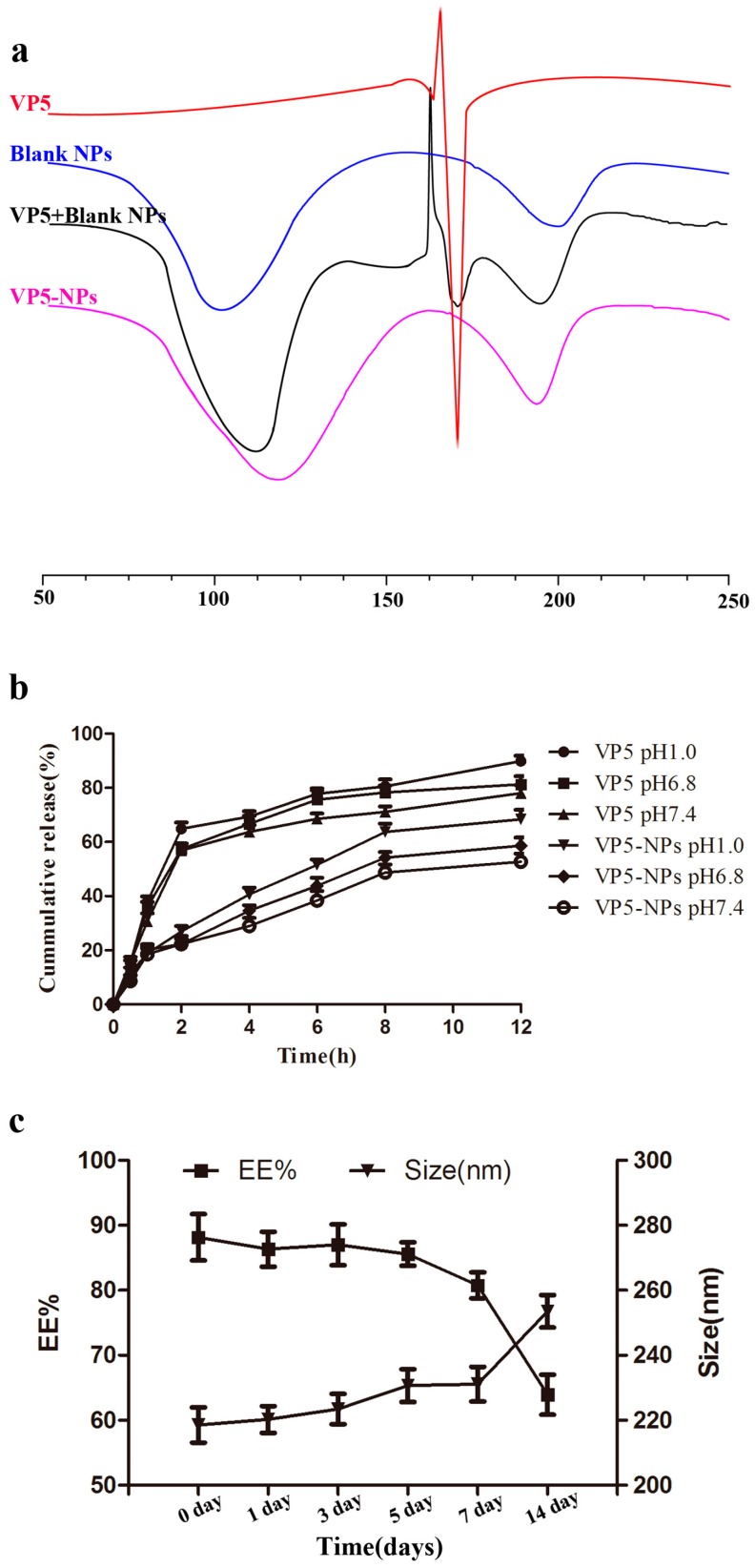
Characterization of VP5-NPs by differential scanning calorimetry (DSC), in vitro release and stability. (**a**) DSC curves of free VP5, blank nanoparticles, the physical mixture of VP5 and blank nanoparticles, and VP5-NPs; (**b**) Release profiles of free VP5 and VP5-NPs in different phosphate buffers (pH 1.0, 6.8 and 7.4); (**c**) Change in size and EE% of VP5-NPs in 2 weeks.

A differential scanning calorimetry (DSC) analysis is shown in Figure 3a, which shows that free VP5 presented an endothermic peak at 167.0 °C followed by an exothermic peak probably due to its degradation. The same endothermic peak was absent in DSC thermogram of VP5-NPs, in which there were only two endothermic peaks at 118.2 and 193.6 °C corresponding to the glass transition temperature (*T*g) of the PLGA and PVA, respectively. However, the physical mixture of VP5 and blank nanoparticles showed three endothermic peaks which corresponded to the free VP5 and the *T*g of the PLGA and PVA.

In vitro release profiles of VP5 from VP5-NPs were presented in Figure 3b. The VP5 loaded in VP5-NPs was released progressively without obvious burst release in all of the release media. The releases of free VP5 and VP5-NPs both exhibited typical pH-dependent releasing behavior. Within a 12 h period, compared with the 78.0% release of free VP5 at pH 7.4, only 52.7% of VP5 was released from VP5-NPs.

VP5-NPs displayed a good stability with no detectable changes in particle sizes, ζ potential or EE% for at least 1 week (Figure 3c), which was benefit from the negatively charges on nanoparticles (Figure 2b). When VP5-NPs were stored beyond 2 weeks, an enlargement of particle size and a drop of EE% of VP5 were observed. Overall, VP5-NPs were suggested to be stable in 4 °C for 1 week and be further investigated to develop a freeze-drying formulation for oral administration.

### 2.3. In Vivo Antihypertensive Efficacy

**Figure 4:**
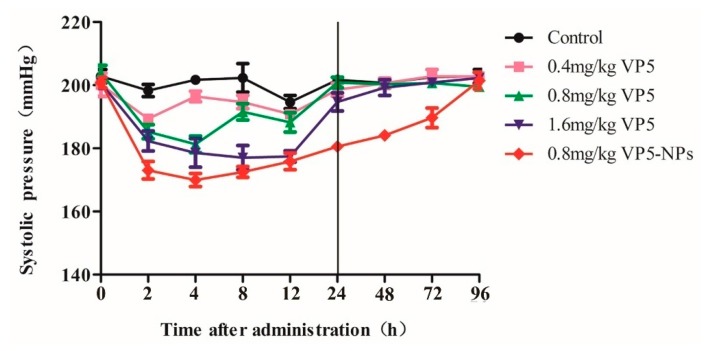
Antihypertensive effects of different doses of VP5 and VP5-NPs in SHRs within 96 h by a single oral administration.

As seen in Figure 4 and Table 1, VP5 exhibited a significant blood pressure-lowering (BPL) effect in a dose-dependent manner. Administration of 0.4 mg/kg of VP5 significantly decreased systolic blood pressure (SBP) by 10.0 mm Hg at 2 h post-administration (*p* < 0.01). VP5 at a dose of 0.8 mg/kg decreased SBP by 13.4 and 20.2 mmHg at 2 and 4 h post-administration, respectively. The decreased SBP between 2 to 12 h post administration was observed after treated by 1.6 mg/kg VP5, and the maximum BPL effect (20.9 mmHg) was at 8 h post administration. SBP returned to the untreated levels within 24 h post-administration in all three groups with variations in time duration. Then, we further investigated the BPL effect of VP5-NPs. 0.8 mg/kg of VP5-NPs strikingly decreased SBP by 26.7 mmHg at 2 h post administration and showed a maximized BPL effect at 4 h by 31.7 mmHg. 4 h post treatment, SBP ascended slowly and smoothly, even after 72 h, significant BPL effect was still observed as a reduction of 18.7 mmHg (*p* < 0.01). SBP returned to the initial level at 96 h post administration.

## 3. Discussion

VP5-NPs were prepared by a simple and controllable double emulsification method [16]. As previously reported [17], entrapment efficiency (EE%) and size are important indexes for evaluating the quality of nanoparticles. High EE% can improve utilization of the drug and smaller size could enhance the absorption of intestinal epithelial cells. Therefore, high EE% and better size were optimized by varying such parameters, including the amount of PLGA, the volume of acetone, pH and the volume of inner aqueous phase, the concentration and the volume of PVA, and the sonication time. Firstly, the influence of the amount of PLGA on EE% and size potentially due to the higher viscosity in the organic phase as the PLGA concentration increased. More viscous organic phase might result in larger droplets and less net shear stress in the emulsion, as well as decreased diffusion of VP5 from inner aqueous to organic phase. Thus, the increasing PLGA amount was shown to result in larger particle size and higher EE% [18,19,20]. Tuning the proportions of dichloromethane and acetone in the organic phase may produce nanoparticles with more desirable properties [21]. According to our preliminary experiments [22], the volume of acetone was optimized to generate VP5-NPs with higher EE% and better particle size, while dichloromethane was fixed. The presence of acetone in the organic phase could reduce the interfacial energy at the oil/water interface and enhance the stability of droplets, which contributed to the smaller sizes of nanoparticles [23]. However, the presence of acetone might enhance the VP5 dispersion from the inner to the outer aqueous phase and cause a sharp decrease in the EE% of VP5 [24]. Due to the effects on solubility [25] and ionizing status of VP5, the pH of the inner aqueous phase was further optimized. The observed tendency probably resulted from the different status of VP5 in the internal phase. When the pH was greater than the isoelectric point (pI) of the VP5 peptide (pI = 6.0), the charge distribution in the peptide could be changed and had an impact on the encapsulation of peptide [26]. The effect of inner aqueous phase volume was also investigated. The increase in W1/O volume ratio was able to enhance the entrapment of the internal phase by the organic phase, which led to the minimal particle size and maximal EE% [27,28]. When W1/O volume ratio exceeded a threshold, the organic phase failed to entrap the internal phase sufficiently and resulted in an increase in the size of droplets and a decrease in the EE%.

In this study, PVA, as the emulsifying agent, was used as a stabilizer during the emulsion formation and to produce stabilized organic droplets [19]. The impacts of the concentration and volume of PVA were then evaluated. The decrease of the particle size was probably caused by the excess amount of PVA residing at organic solvent/aqueous interface, and reduced the surface tension [29,30,31]. However, the increase in PVA concentration enhanced the outer aqueous viscosity [19], which impeded the diffusion of VP5 in the inner aqueous phase into outside and thereby improved the incorporation. The increase of the volume of PVA could enhance the net sheer stress and resulted in smaller size of particles [32]. On the other hand, the viscosity in the external phase could increase with higher PVA volume, which led to a bigger particle size. The EE% could be maximized when the interaction of primary emulsion with external phase was optimal. Peptides are generally subjected to denaturation under various stress conditions, such as sonication and homogenization [33]. Hence, the sonication duration was expected to be critical for the VP5-NP preparation. The short W1/O sonication time was suggested to cause VP5 incompletely being entrapped in the primary emulsion and lower down the EE%. Longer sonication time was speculated to break the coarse emulsion drops into nanodroplets, which could cause the observed particle size of VP5-NPs and the improved EE% [34]. However, when the formation of primary emulsion was good, longer sonication time would lead to the leakage of VP5 from the internal phase to the external phase, causing a decrease in EE%. Similar to the effect observed with W1/O, prolonged W1/O/W2 sonication time could enhance the EE%, and it could lead to an increasing net sheer force and result in a decreasing particle size at the same time.

To investigate the existing state of VP5 and verify the successful entrapment of VP5 into nanoparticles, DSC analysis was performed [35]. The *T*g of PVA was in agreement with the previous research [18], but that of PLGA was higher than that reported in the literature most probably due to the different ratio of lactic acid / glycolic acid (LA:GA) and molecular weight of PLGA [36].

The release of VP5 out of nanoparticles was dramatically lower than the unincorporated VP5 when the same dialysis bag was used, although there might be some interaction of VP5 itself with the applied dialysis. Theoretically, the dialysis bag for the in vitro release study should let the free agent with small molecular weight get through freely. However, many dialysis bags would influence the passage of the agents investigated, probably because of some interaction including adsorption or hydrophobic interaction [37]. Only about 90% of the free VP5 can diffuse into the dispersed medium after 12 h, thus other methods for the in vitro release test of VP5 need to be developed in the future. The in vitro sustained release in all of the release media indicated a prolonged release of VP5-NPs and might lead to a potent and prolonged therapeutic efficacy of VP5-NPs. The pH-dependent releasing behavior was most probably caused by the degradation of PLGA [38]. The release percentages in media with different pH may provide some evidence that VP5-NPs could provide sustainable release of VP5 in both stomach and intestine and protect VP5 from premature degradation [39].

It is recognized that oral delivery of peptide has been a great continuous challenge due to its poor stability in the GI tract and low permeability through the intestinal epithelium membrane [40,41,42]. Numerous promising strategies have been utilized to circumvent these obstacles [43], including enzyme inhibitors [44] and solid lipid NPs [45] to protect these drugs from enzymatic degradation. Chemical absorption enhancers [46] were also used to improve intestinal epithelium membrane permeability. However, these approaches had only achieved limited success so far.

PLGANPs exhibit a wide range of erosion times, tunable biodegradation and mechanical properties [47] and have been widely used in academic and industrial research [7,10,11]. Here, we presented the encapsulation of VP5 into PLGANPs, which could expect to enhance the stability of VP5 and prolong the active ingredient release for a better therapeutic efficacy. In our study, VP5-NPs exhibited a four-time prolonged BPL effect than high-dose of free drug (Figure 4) that was partially supported by the in vitro sustained release experiment (Figure 3c). Moreover, VP5-NPs of small size could enhance the intestinal absorption and subsequently reach the systemic circulation and gradually release VP5, which might contribute a long-term antihypertensive effect [48]. It was previously reported PLGA is not a gastro-resistant polymer [49], which presented some conflicts with our results. The inconsistent observation could be explained by the incomplete degradation of VP5-NPs, where the remaining intact nanoparticles could still proceed with the fluid to the small intestine and be uptaken by the intestinal tissue. Another possible explanation could be the short VP5 oligopeptide being resistant to the digestion of gastrointestinal juices [50]. Even if being released from nanoparticles and losing shields, intact VP5 could be absorbed by intestinal epithelial cells to produce a response.

Currently, the mechanism of enhancement in therapeutic efficacy is still under investigation. The efficiency of VP5 being absorbed into systemic circulation requires further quantification. The association between elevated therapeutic efficacy and the area under concentration-time curve (AUC) or bioavailability needs further elucidation.

Overall, we systematically optimized the factors that could affect the features of NPs, and successfully constructed VP5-NPs with a high entrapment efficiency and small particle size. Then, we evaluated its antihypertensive efficiencies in vivo, including the crude VP5 and VP5-NPs, found out that VP5 decreased blood pressure in a dose dependent manner. Much to our surprise, a medium dose of VP5-NPs showed stronger BPL effect than a high dose of crude VP5, and maintained a prolonged BPL effect even up to 72 h. This may be partly explained by the controlled release of VP5 and the protection effect of the nanoparticle shield avoiding enzymatic degradatio, and subsequently elevating the bioavailability of VP5. This may provide some methodological clues and insights for the therapy of hypertension.

## 4. Materials and Methods

### 4.1. Materials

VP5 (purity > 99%) was purchased from Phtdpeptides Co., Ltd. (Zhengzhou, China). PLGA (*M*_W_ = 15 kDa; LA/GA = 75:25) was purchased from Jinan Daigang Biomaterial Co., Ltd. (Jinan, China). Poly (vinyl alcohol) (PVA, *M*_W_ = 30–70 kDa; HD, 80%) was procured from Sigma-Aldrich (St. Louis, MO, USA). All other reagents were of analytical grade and were used as supplied.

40-week-old male spontaneously hypertensive rats (SHRs) weighing between 350 to 400 g were purchased from Beijing Vital River Laboratory Animal Technology Co., Ltd. (Beijing, China). The trial was approved by the Animal experimental ethics committee of State Key Laboratory of Biotherapy of Sichuan University (20150356, 28 December 2015).

### 4.2. Preparation of VP5-NPs

VP5-NPs were prepared by a double-emulsion (W1/O/W2) solvent evaporation method. Briefly, VP5 was dissolved in deionized water to form the inner aqueous phase. The organic phase containing PLGA was emulsified with the inner aqueous phase by probe sonication in ice bath to form the primary W1/O emulsion. The obtained primary emulsion was then added to PVA solution and further sonicated to gain the final W1/O/W2 double emulsion. The organic phase was rapidly removed by evaporation under vacuum at 37 °C. The VP5-NPs were obtained through ultracentrifugation at a speed of 50,000 rpm for 1 h to remove PVA and the unencapsulated VP5. The blank nanoparticles were also prepared using the similar process.

### 4.3. Characterization of VP5-NPs

#### 4.3.1. Particle Size and ζ Potential

The mean particle size, size distribution and ζ potential were measured using a Zetasizer (Zetasizer Nano-ZS 90; Malvern Instruments Ltd., Malvern, UK) at 25 °C. The prepared VP5-NPs were dispersed in deionized water with 10 times and experiments were conducted in triplicate. All the data were presented as mean ± SD.

#### 4.3.2. Entrapment Efficiency and Drug Loading

The supernatant after centrifuging the colloidal solution during the preparation of VP5-NPs was used to determine EE% and DL% of VP5-NPs as previously described [51] with minor modifications. Briefly, the amount of untrapped VP5 in supernatant was calculated by high performance liquid chromatography (HPLC, Waters Alliance 2695). A reverse-phase C18 column (150 mm × 4.6 mm, pore size 5 µm, Cosmosil, Nacalai, Japan) was used for the chromatographic separation with a mobile phase consisting of a mixture of acetonitrile/0.1% TFA water (75/25, *v*/*v*) at a flow rate of 0.8 mL/min. VP5 was detected at a wavelength of 220 nm. EE% and DL% of VP5 were calculated by the following formula:

$$\mathrm{EE}\%\;=\;\left(\frac{\mathrm{Total}\;\mathrm{VP}5\;\mathrm{amount}\;-\;\mathrm{the}\;\mathrm{amount}\;\mathrm{of}\;\mathrm{VP}5\;\mathrm{in}\;\mathrm{supernatant}}{\mathrm{Total}\;\mathrm{VP}5\;\mathrm{amount}}\right)\;\times \;100$$

$$\mathrm{DL}\%\;=\;\left(\frac{\mathrm{Total}\;\mathrm{VP}5\;\mathrm{amount}\;-\;\mathrm{the}\;\mathrm{amount}\;\mathrm{of}\;\mathrm{VP}5\;\mathrm{in}\;\mathrm{supernatant}}{\mathrm{Total}\;\mathrm{weight}\;\mathrm{of}\;\mathrm{nanoparticles}}\right)\;\times \;100$$

#### 4.3.3. Appearance

The morphology of the VP5-NPs was examined by transmission electronic microscopy (TEM, H-600, Hitachi, Japan). Before analysis, the prepared samples were diluted with deionized (DI) water and negatively stained with 2 wt % phosphotungstic acid solution for 30 s, and then they were placed on a copper electron microscopy grids with a thin film for observation.

#### 4.3.4. Differential Scanning Calorimetry (DSC)

The physical state of VP5 loaded in VP5-NPs was investigated by differential scanning calorimetry (DSC, 200PC, Netzsch, Karlsruhe, Germany) under nitrogen atmosphere at a flow rate of 20 mL/min. Freeze-dried VP5-NPs, blank nanoparticles, VP5 and the physical mixture of the latter two samples with the same mass ratio as those in VP5-NPs were heated from 50 to 250 °C at speed of 10 °C/min.

#### 4.3.5. In Vitro Release

The in vitro release profiles of VP5 from VP5-NPs were carried out in PBS buffer at physiological pH condition (pH 7.4), in simulated intestinal fluid (pH 6.8), and in simulated gastric fluid (pH 1.0) by the dialysis method. Briefly, both VP5-NPs and free VP5 solution were first dispersed in release media for dialysis (MWCO 3000), which were shaken at 37 °C with a speed of 100 rpm. At a given time point, 1 mL buffer was removed and replaced by another 1 mL fresh release medium. The content of VP5 was measured by HPLC as described in the Section 4.3.2.

#### 4.3.6. Stability

VP5-NPs were stored at 4 °C for two weeks to investigate the preliminary stability. The particle size, size distribution, and EE% of VP5 were measured as described above and the changes over time was analyzed.

### 4.4. In Vivo Antihypertensive Efficacy

The in vivo pharmacodynamics studies were performed using SHRs, which were allowed for one-week acclimatization in independent cages. They were given free access to commercial laboratory feed (MF; Beijing HFK Bioscience Co., Beijing, China) and tap water in a controlled temperature room (1–22 °C) with a 12 h light-dark cycle. Four groups of SHRs, 6 rats each, were administered a single oral dose of crude drug VP5 or VP5-NPs. The crude drug VP5 was dissolved in saline, and the control group received only saline. Then, the dosing volume was calculated from the bodyweight of SHRs. The change in blood pressure caused by different dosage was indirectly recorded using the tail cuff method (Softron BP-2010A; Softron Beijing Biotechnology, Beijing, China) at 0, 2, 4, 8, 12, 24 h and every one day after administration.

### 4.5. Statistical Analysis

The statistical analysis was performed using the Statistical Product and Service Solutions software (SPSS V19.0, IBM Corp., New York, NY, USA). Data were analyzed by one-way analysis of variance. *p* < 0.05 was considered a statistically difference, and *p* < 0.01 was considered a statistically significant difference.

## 5. Conclusions

PLGANPs loaded with the antihypertensive small peptide model VP5 were successfully prepared using a simply double-emulsion solvent evaporation method. The optimal VP5-NPs showed desirable pharmaceutical properties through systematical process-parameter investigation, including small size, high EE% and sustained release. Moreover, VP5-NPs exhibited an enhanced antihypertensive function with a longer duration, up to 96 h in SHRs. Altogether, PLGANPs can serve as a compelling strategy for oral delivery of antihypertensive small peptides, and VP5-NPs might be a potential strategy for hypertension treatment in the future.

## Figures and Tables

**Table 1:** The decreasing amplitude of systolic blood pressure of spontaneously hypertensive rats (SHRs) at different time points after a single oral dose (X ± SD, *n* = 6) (mmHg).

Time	Group Control				
	Saline Group	0.4 mg/kg VP5	0.8 mg/kg VP5	1.6 mg/kg VP5	0.8 mg/kg VP5-NPs
2 h	4.5 ± 3.2	10.0 ± 1.3 ***	13.4 ± 2.2 ***	17.7 ± 3.8 ***	26.7 ± 3.7 ***
4 h	1.2 ± 0.6	5.2 ± 1.9 *	20.2 ± 3.6 ***	18.5 ± 5.8 ***	31.7 ± 2.3 ***
8 h	1.0 ± 4.7	8.2 ± 2.6	12.4 ± 2.3	20.9 ± 4.2 ***	28.9 ± 1.9 ***
12 h	8.2 ± 1.2	9.4 ± 1.3	15.8 ± 3.7	19.5 ± 3.0 ***	23.6 ± 3.8 ***
24 h	1.2 ± 1.5	1.6 ± 1.6	3.5 ± 0.8	6.0 ± 3.4	21.5 ± 0.7 ***
48 h	2.2 ± 1.6	−0.5 ± 1.8	2.4 ± 1.2	1.5 ± 2.5	20.4 ± 1.0 ***
72 h	0.3 ± 1.5	−2.7 ± 1.1	3.2 ± 1.6	−0.1 ± 1.0	18.7 ± 3.9 ***
96 h	0.6 ± 0.5	−2.6 ± 2.7	4.4 ± 0.7	−1.6 ± 1.4	−0.6 ± 3.5

One-way analysis of variance was used for calculating statistical significance, which was set at * *p* < 0.05, *** *p* < 0.01. VP5-NPs and three crude VP5 groups versus control group.

## Abbreviations

ACE	Angiotensin converting enzyme
VP5	Val-Leu-Pro-Val-Pro
RAAS	Renin-angiotensin-aldosterone system
ARB	Angiotensin receptor blocker
SBP	Systolic blood pressure
DBP	Diastolic blood pressure
IPP	Ile-Pro-Pro
VPP	Val-Pro-Pro
GI	Gastrointestinal
BPL	Blood pressure-lowering
